# New approximation inequalities for circular functions

**DOI:** 10.1186/s13660-018-1910-9

**Published:** 2018-11-16

**Authors:** Ling Zhu, Marija Nenezić

**Affiliations:** 1 Department of Mathematics, Zhejiang Gongshang University, Hangzhou City, China; 2 School of Electrical Engineering, University of Belgrade, Belgrade, Serbia

**Keywords:** 26D05, 26D15, 33B10, Circular functions, Bernoulli numbers, Mitrinovic–Adamovic inequality, Exponential approximation inequalities

## Abstract

In this paper, we obtain some improved exponential approximation inequalities for the functions $(\sin x)/x$ and $\sec(x)$, and we prove them by using the properties of Bernoulli numbers and new criteria for the monotonicity of quotient of two power series.

## Introduction

The following result is known as the Mitrinovic–Adamovic inequality [1, 2]:

1.1 $$ \biggl( \frac{\sin x}{x} \biggr) ^{3}>\cos x,\quad 0< x< \frac{\pi }{2}. $$

Nishizawa [3] gave the upper bound of the function $( ( \sin x ) /x ) ^{3}$ in the form of the above inequality (1.1) and obtained the following power exponential inequality:

1.2 $$ \biggl( \frac{\sin x}{x} \biggr) ^{3}< ( \cos x ) ^{1-2x/ \pi }, \quad 0< x< \frac{\pi}{2}. $$

Chen and Sándor [4] looked into the bounds for the function sec*x* and obtain the following result for $0< x<\pi /2$:

1.3 $$ \frac{\pi^{2}}{\pi^{2}-4x^{2}}< \sec x< \frac{4\pi }{\pi^{2}-4x^{2}}. $$

Nishizawa [3] obtained the following inequality with power exponential functions derived from the right-hand inequality side of (1.3):

1.4 $$ \biggl( \frac{4\pi }{\pi^{2}-4x^{2}} \biggr) ^{4x^{2}/\pi^{2}}< \sec x,\quad 0< x< \frac{ \pi }{2}. $$

The purpose of this article is to establish some exponential approximation inequalities which improve the ones of (1.1)–(1.4). We prove these results for circular functions by using the properties of Bernoulli numbers and new criteria for the monotonicity of quotient of two power series.

### Theorem 1.1

*Let* $0< x<\pi /2$, $a=2/15\thickapprox 0.13333$ *and* $b=4/\pi^{2}\thickapprox 0.40528$. *Then we have*

1.5 $$ ( \cos x ) ^{1-ax^{2}}< \biggl( \frac{\sin x}{x} \biggr) ^{3}< ( \cos x ) ^{1-bx^{2}}, $$

*where* *a* *and* *b* *are the best constants in* (1.5).

### Theorem 1.2

*Let* $0< x<\pi /2$, $c=19/945\thickapprox 0.02011$ *and* $d=8(30-\pi^{2})/(15 \pi^{4})\thickapprox 0.11022$. *Then we have*

1.6 $$ ( \cos x ) ^{1-2x^{2}/15-cx^{4}}< \biggl( \frac{\sin x}{x} \biggr) ^{3}< ( \cos x ) ^{1-2x^{2}/15-dx ^{4}}, $$

*where* *c* *and* *d* *are the best constants in* (1.6).

### Theorem 1.3

*Let* $0< x<\pi /2$, $b=4/\pi^{2}\thickapprox 0.40528$ *and* $p=1/(2\ln (4/ \pi ))\thickapprox 2.0698$. *Then we have*

1.7 $$ \biggl( \frac{4\pi }{\pi^{2}-4x^{2}} \biggr) ^{bx^{2}}< \sec x< \biggl( \frac{4 \pi }{\pi^{2}-4x^{2}} \biggr) ^{px^{2}}, $$

*where* *b* *and* *p* *are the best constants in* (1.7).

### Theorem 1.4

*Let* $0< x<\pi /2$,

$$ \alpha =\frac{1}{12\ln \frac{4}{\pi }}-\frac{2}{\pi^{2}\ln^{2}\frac{4}{ \pi }}\thickapprox -3.1277,\quad\quad \beta = \frac{16}{\pi^{4}} \biggl( 1- \frac{1}{8}\frac{\pi^{2}}{\ln \frac{4}{\pi }} \biggr) \thickapprox -0.67462. $$

*Then we have*

1.8 $$ \biggl( \frac{4\pi }{\pi^{2}-4x^{2}} \biggr) ^{x^{2}/ ( 2\ln ( 4/\pi ) ) +\alpha x^{4}}< \sec x< \biggl( \frac{4\pi }{\pi ^{2}-4x^{2}} \biggr) ^{x^{2}/ ( 2\ln ( 4/\pi ) ) + \beta x^{4}}, $$

*where* *α* *and* *β* *are the best constants in* (1.8).

We note that the right-hand side of the inequality (1.5) is stronger than that one in (1.2) due to

$$ 1-\frac{4}{\pi^{2}}x^{2}= \biggl( 1+\frac{2x}{\pi } \biggr) \biggl( 1-\frac{2x}{ \pi } \biggr) >1-\frac{2x}{\pi } $$

while the double inequality (1.6) and (1.8) are sharper than the (1.5) and (1.7), respectively.

## Lemmas

### Lemma 2.1

([5–8])

*Let* $B_{2n}$ *be the even*-*indexed Bernoulli numbers*, $n=1,2,\ldots $ . *Then*

2.1 $$\begin{aligned}& \frac{2(2n)!}{(2\pi )^{2n}}\frac{2^{2n}}{2^{2n}-1}< \vert B_{2n} \vert < \frac{2(2n)!}{(2 \pi )^{2n}}\frac{2^{2n}}{2^{2n}-2}, \end{aligned}$$

2.2 $$\begin{aligned}& \frac{2^{2n-1}-1}{2^{2n+1}-1}\frac{(2n+2)(2n+1)}{\pi^{2}}< \frac{ \vert B _{2n+2} \vert }{ \vert B_{2n} \vert }< \frac{2^{2n}-1}{2^{2n+2}-1} \frac{(2n+2)(2n+1)}{\pi ^{2}}. \end{aligned}$$

### Lemma 2.2

*Let* $B_{2n}$ *be the even*-*indexed Bernoulli numbers*. *Then the following power series expansion*:

2.3 $$ \ln \frac{\sin x}{x}=-\sum_{n=1}^{\infty } \frac{2^{2n}}{2n ( 2n ) !} \vert B_{2n} \vert x^{2n},\quad 0< \vert x \vert < \pi , $$

*and*

2.4 $$ \ln \cos x=-\sum_{n=1}^{\infty } \frac{2^{2n}-1}{2n ( 2n ) !}2^{2n} \vert B _{2n} \vert x^{2n}, \quad \vert x \vert < \frac{\pi }{2}, $$

*hold*.

### Proof

The following power series expansions can be found in [9, 1.3.1.4(2)(3)]:

2.5 $$\begin{aligned}& \cot x=\frac{1}{x}-\sum_{n=1}^{\infty } \frac{2^{2n}}{ ( 2n ) !} \vert B_{2n} \vert x^{2n-1}, \end{aligned}$$

2.6 $$\begin{aligned}& \tan x=\sum_{n=1}^{\infty }\frac{2^{2n}-1}{ ( 2n ) !}2^{2n} \vert B _{2n} \vert x^{2n-1}. \end{aligned}$$

By (2.5) and (2.6) we have

$$\begin{aligned} \ln \frac{\sin x}{x} =& \int_{0}^{x} \biggl( \ln \frac{\sin t}{t} \biggr) ^{\prime }\,dt= \int_{0}^{x} \biggl( \cot t-\frac{1}{t} \biggr) \,dt \\ =&-\sum_{n=1}^{\infty }\frac{2^{2n}}{2n ( 2n ) !} \vert B_{2n} \vert x ^{2n} \end{aligned}$$

and

$$\begin{aligned} \ln \cos x =& \int_{0}^{x} ( \ln \cos t ) ^{\prime }\,dt=- \int _{0}^{x}\tan t\,dt \\ =&-\sum_{n=1}^{\infty }\frac{2^{2n}-1}{2n ( 2n ) !}2^{2n} \vert B _{2n} \vert x^{2n}. \end{aligned}$$

 □

### Lemma 2.3

([10])

*Let* $a_{n}$ *and* $b_{n}$ ($n=0,1,2,\ldots $) *be real numbers*, *and let the power series* $A(t)=\sum_{n=0}^{\infty }a_{n}t^{n}$ *and* $B(t)=\sum_{n=0} ^{\infty }b_{n}t^{n}$ *be convergent for* $\vert t \vert < R$ ($R\leq +\infty $). *If* $b_{n}>0$ *for* $n=0,1,2,\ldots $ , *and if* $\varepsilon_{n}=a_{n}/b_{n}$ *is strictly increasing* (*or decreasing*) *for* $n=0,1,2,\ldots $ , *then the function* $A(t)/B(t)$ *is strictly increasing* (*or decreasing*) *on* $(0,R)$ ($R\leq +\infty $).

In order to prove Theorem 1.4, we need the following lemma. We introduce a useful auxiliary function $H_{f,g}$. For $-\infty \leq a< b\leq \infty $, let *f* and *g* be differentiable on $(a,b)$ and $g^{\prime }\neq 0$ on $(a,b)$. Then the function $H_{f,g}$ is defined by

$$ H_{f,g}=\frac{f^{\prime }}{g^{\prime }}g-f. $$

The function $H_{f,g}$ has some good properties and plays an important role in the proof of a monotonicity criterion for the quotient of power series.

### Lemma 2.4

([11])

*Let* $A ( t ) =\sum_{k=0}^{\infty }a_{k}t^{k}$ *and* $B ( t ) =\sum_{k=0}^{\infty }b_{k}t^{k}$ *be two real power series converging on* $( -r,r ) $ *and* $b_{k}>0$ *for all k*. *Suppose that*, *for certain* $m\in N$, *the non*-*constant sequence* $\{ a_{k}/b _{k} \} $ *is increasing* (*resp*. *decreasing*) *for* $0\leq k\leq m$ *and decreasing* (*resp*. *increasing*) *for* ${k\geq m}$. *Then the function* $A/B$ *is strictly increasing* (*resp*. *decreasing*) *on* $( 0,r ) $ *if and only if* $H_{A,B} ( r^{-} ) \geq \textit{(resp. }{\leq} \textit{) }0$. *Moreover*, *if* $H_{A,B} ( r^{-} ) <\textit{(resp. }{>}\textit{) }0$, *then there exists* $t_{0}\in ( 0,r ) $ *such that the function* $A/B$ *is strictly increasing* (*resp*. *decreasing*) *on* $( 0,t_{0} ) $ *and strictly decreasing* (*resp*. *increasing*) *on* $( t_{0},r ) $.

## Proof of Theorem 1.1

Let

$$ F_{1}(x)=\frac{\frac{3\ln \frac{\sin x}{x}}{\ln \cos x}- 1}{x^{2}}=\frac{3\ln \frac{\sin x}{x}-\ln \cos x}{x^{2}\ln \cos x}=\frac{ \ln \cos x-3\ln \frac{\sin x}{x}}{-x^{2}\ln \cos x}= \frac{A(x)}{B(x)},\quad 0< x< \frac{ \pi }{2}, $$

where

$$\begin{aligned} A(x) =&\ln \cos x-3\ln \frac{\sin x}{x}=-\sum_{n=1}^{\infty } \frac{2^{2n}-1}{2n ( 2n ) !}2^{2n} \vert B_{2n} \vert x^{2n}+\sum_{n=1}^{\infty } \frac{3 \cdot 2^{2n}}{2n ( 2n ) !} \vert B_{2n} \vert x^{2n} \\ =&-\sum_{n=1}^{\infty }\frac{2^{2n}-4}{2n ( 2n ) !}2^{2n} \vert B _{2n} \vert x^{2n}=-\sum _{n=2}^{\infty }\frac{2^{2n}-4}{2n ( 2n ) !}2^{2n} \vert B _{2n} \vert x^{2n} \\ =&-\sum_{n=1}^{\infty }\frac{2^{2n+2}-4}{(2n+2) ( 2n+2 ) !}2^{2n+2} \vert B _{2n+2} \vert x^{2n+2}=\sum _{n=1}^{\infty }a_{n}x^{2n} \end{aligned}$$

and

$$ B(x)=-x^{2}\ln \cos x=\sum_{n=1}^{\infty } \frac{2^{2n}-1}{2n ( 2n ) !}2^{2n} \vert B_{2n} \vert x^{2n+2}=\sum_{n=1}^{\infty }b_{n}x^{2n} $$

by Lemma 2.2. Let

$$ \frac{a_{n}}{b_{n}}=-\frac{16n}{ ( 2n+2 ) (2n+1) ( n+1 ) }\frac{ \vert B_{2n+2} \vert }{ \vert B_{2n} \vert }=-e_{n}, $$

where

$$ e_{n}=\frac{16n}{ ( 2n+2 ) (2n+1) ( n+1 ) }\frac{ \vert B _{2n+2} \vert }{ \vert B_{2n} \vert }. $$

We now show that $\{ e_{n} \} $ is increasing for $n\geq 1$. Since

$$\begin{aligned}& e_{n-1} =\frac{16(n-1)}{ ( 2n ) (2n-1) ( n ) }\frac{ \vert B _{2n} \vert }{ \vert B_{2n-2} \vert }< \frac{16(n-1)}{ ( 2n ) (2n-1) ( n ) } \frac{1}{ ( 2\pi ) ^{2}}\frac{2n ( 2n-1 ) 2^{2n-1}}{2^{2n-1}-1}, \\& e_{n} >\frac{16n}{ ( 2n+2 ) (2n+1) ( n+1 ) }\frac{1}{ ( 2\pi ) ^{2}}\frac{(2n+2) ( 2n+1 ) ( 2^{2n-1}-1 ) }{2^{2n-1}} \end{aligned}$$

by Lemma 2.1, the proof of $e_{n-1}< e_{n}$ for $n\geq 2$ can be completed when proving

$$ \frac{n}{n+1}\frac{ ( 2^{2n-1}-1 ) }{2^{2n-1}}>\frac{n-1}{n} \frac{2^{2n-1}}{2^{2n-1}-1}. $$

In fact,

$$ n^{2} \bigl( 2^{2n-1}-1 \bigr) ^{2}- \bigl(n^{2}-1\bigr)2^{2(2n-1)}=2^{4n-2}-4^{n}n ^{2}+n^{2} >0 $$

for $n\geq 2$. So $\{ a_{n}/b_{n} \} _{n\geq 1}$ is decreasing, and $F_{1}(x)$ is decreasing on $(0,\pi /2)$ by Lemma 2.3. In view of $F_{1}(0^{+})=-2/15$, and $F_{1}((\pi /2)^{-})=-4/ \pi^{2}$, the proof of Theorem 1.1 is complete.

## Proof of Theorem 1.2

(i) We first prove the left-hand side inequality of (1.6). Let

$$ F_{2}(x)=3\ln \frac{\sin x}{x}- \biggl( 1-\frac{2}{15}x^{2}- \frac{19}{945}x^{4} \biggr) \ln \cos x, \quad 0< x< \frac{\pi }{2}. $$

Then by Lemma 2.2 we have

$$ F_{2}(x)=\sum_{n=3}^{\infty }i_{n}2^{2n-2} \vert B_{2n} \vert x^{2n+2}, $$

where

$$ i_{n}=\frac{16(2^{2n+2}-4)}{(2n+2) ( 2n+2 ) !}\frac{ \vert B_{2n+2} \vert }{ \vert B _{2n} \vert }-\frac{8 ( 2^{2n}-1 ) }{30n ( 2n ) !}- \frac{19 ( 2^{2n-2}-1 ) }{945(2n-2) ( 2n-2 ) !}\frac{ \vert B_{2n-2} \vert }{ \vert B _{2n} \vert }. $$

By Lemma 2.1, we have

$$\begin{aligned} i_{n} >&\frac{16(2^{2n+2}-4)}{(2n+2) ( 2n+2 ) !}\frac{(2n+2)(2n+1)(2^{2n-1}-1)}{ \pi^{2}(2^{2n+1}-1)}-\frac{8 ( 2^{2n}-1 ) }{30n ( 2n ) !} \\ & {} -\frac{19 ( 2^{2n-2}-1 ) }{945(2n-2) ( 2n-2 ) !}\frac{ \pi^{2}(2^{2n-1}-1)}{(2n)(2n-1)(2^{2n-3}-1)} \\ =&\frac{1}{ ( 2n ) !}j_{n} \end{aligned}$$

with

$$\begin{aligned} j_{n} =&\frac{16(2^{2n+2}-4)}{(2n+2)} \frac{(2^{2n-1}-1)}{\pi^{2}(2^{2n+1}-1)}-\frac{8}{15} \frac{2^{2n}-1}{2n}-\frac{19}{945}\frac{2^{2n-2}-1}{(2n-2)}\frac{\pi ^{2}(2^{2n-1}-1)}{(2^{2n-3}-1)} \\ >&\frac{16(2^{2n+2}-4)}{(2n+2)} \frac{(2^{2n-1}-1)}{\frac{79}{8}(2^{2n+1}-1)}-\frac{8}{15} \frac{(2^{2n}-1)}{2n}- \frac{19}{945}\cdot \frac{79}{8} \frac{(2^{2n-2}-1)}{(2n-2)}\frac{(2^{2n-1}-1)}{(2^{2n-3}-1)} \\ =&\frac{1}{1{,}194{,}480}\frac{h(n)}{n ( 2\cdot 2^{2n}-1 ) ( 2^{2n}-8 ) ( n-1 ) ( n+1 ) } \end{aligned}$$

due to $\pi^{2}<79/8$, where

$$\begin{aligned} h(n) =&\bigl(1{,}061{,}146n^{2}-2{,}172{,}518n+637{,}056 \bigr)2^{6n} \\ &{}-\bigl(13{,}695{,}401n^{2}-22{,}830{,}487n+6{,}052{,}032 \bigr)2^{4n} \\ & {} +\bigl(39{,}747{,}422n^{2}-52{,}928{,}098n+7{,}963{,}200 \bigr)2^{2n} \\ &{}-\bigl(27{,}468{,}904n ^{2}-31{,}914{,}392n+2{,}548{,}224 \bigr) \\ =&2^{4n}h_{1}(n)+h_{2}(n). \end{aligned}$$

It is not difficult to verify

$$\begin{aligned} h_{1}(n) =&\bigl(1{,}061{,}146n^{2}-2{,}172{,}518n+637{,}056 \bigr)2^{2n} \\ &{}-\bigl(13{,}695{,}401n ^{2}-22{,}830{,}487n+6{,}052{,}032 \bigr) \\ >&0 \end{aligned}$$

and

$$\begin{aligned} h_{2}(n) =&\bigl(39{,}747{,}422n^{2}-52{,}928{,}098n+7{,}963{,}200 \bigr)2^{2n} \\ &{}-\bigl(27{,}468{,}904n^{2}-31{,}914{,}392n+2{,}548{,}224 \bigr) \\ >&0 \end{aligned}$$

for $n\geq 3$. So $i_{n}>0$ for $n\geq 3$, and $F_{2}(x)>0$ for $x \in (0,\pi /2)$.

(ii) Then we prove the right-hand side inequality of (1.6). Let

$$ F_{3}(x)=3\ln \frac{\sin x}{x}- \biggl( 1-\frac{2}{15}x^{2}- \frac{8}{15}\frac{30-\pi^{2}}{\pi^{4}}x^{4} \biggr) \ln \cos x, \quad 0< x< \frac{\pi}{2}. $$

Then by Lemma 2.2 we have

$$ F_{3}(x)=\sum_{n=2}^{\infty }l_{n}2^{2n-2} \vert B_{2n} \vert x^{2n+2}, $$

where

$$ l_{n}=\frac{16(2^{2n+2}-4)}{(2n+2) ( 2n+2 ) !}\frac{ \vert B_{2n+2} \vert }{ \vert B _{2n} \vert }-\frac{8}{15} \frac{2^{2n}-1}{2n ( 2n ) !}- \frac{8}{15}\frac{30-\pi^{2}}{\pi^{4}}\frac{2^{2n-2}-1}{(2n-2) ( 2n-2 ) !} \frac{ \vert B_{2n-2} \vert }{ \vert B_{2n} \vert }. $$

By Lemma 2.1 we have

$$\begin{aligned} l_{n} < &\frac{16(2^{2n+2}-4)}{(2n+2) ( 2n+2 ) !} \frac{2^{2n}-1}{2^{2n+2}-1}\frac{(2n+2)(2n+1)}{\pi^{2}}- \frac{8}{15}\frac{2^{2n}-1}{2n ( 2n ) !} \\ & {} -\frac{8}{15}\frac{30-\pi^{2}}{\pi^{4}}\frac{2^{2n-2}-1}{(2n-2) ( 2n-2 ) !}\frac{\pi^{2}(2^{2n}-1)}{(2n)(2n-1)(2^{2n-2}-1)}, \end{aligned}$$

that is,

$$\begin{aligned} ( 2n ) !l_{n} < &\frac{16(2^{2n+2}-4)}{(2n+2)}\frac{(2^{2n}-1)}{ \pi^{2}(2^{2n+2}-1)}- \frac{8}{15}\frac{2^{2n}-1}{2n}-\frac{8}{15}\frac{30- \pi^{2}}{\pi^{4}} \frac{2^{2n-2}-1}{(2n-2)} \frac{\pi^{2}(2^{2n}-1)}{(2^{2n-2}-1)} \\ =& \frac{4}{15} \bigl( 2^{2n}-1 \bigr) \frac{t(n)}{\pi^{2}n ( n^{2}-1 ) ( 4\cdot 2^{2n}-1 ) }, \end{aligned}$$

where

$$ t(n)=-\bigl(240n-4\pi^{2}n-4\pi^{2}\bigr)2^{2n}- \bigl(90n^{2}-\bigl(150-\pi^{2}\bigr)n+\pi^{2} \bigr)< 0 $$

for $n\geq 2$. So $l_{n}<0$ for $n\geq 2$ and $F_{3}(x)<0$ for $x \in (0,\pi /2)$.

(iii) Let

$$ F_{4}(x)=\frac{\frac{3\ln \frac{\sin x}{x}}{\ln \cos x}- ( 1- \frac{2}{15}x^{2} ) }{x^{4}},\quad 0< x< \frac{\pi }{2}. $$

Then

$$ F_{4}\bigl(0^{+}\bigr)= -\frac{19}{945},\quad\quad F_{4} \biggl( \biggl( \frac{\pi }{2} \biggr) ^{-} \biggr) =- \frac{8}{15}\frac{30-\pi^{2}}{\pi^{4}}. $$

This complete the proof of Theorem 1.2.

## Proof of Theorem 1.3

(1) Let

$$ G_{1}(x)=\ln \sec x- \biggl( \frac{2x}{\pi } \biggr) ^{2}\ln \frac{4 \pi }{\pi^{2}-4x^{2}},\quad 0< x< \frac{\pi }{2}. $$

Then we get

$$ G_{1}(x)=\sum_{n=0}^{\infty }k_{n}x^{2n+2}, $$

where

$$\begin{aligned}& k_{0} =\frac{1}{2}-\frac{4}{\pi^{2}}\ln \frac{4}{\pi }>0, \\& k_{n} =- \biggl( \biggl( \frac{2}{\pi } \biggr) ^{2n+2} \frac{1}{n}-\frac{2^{2n+2}-1}{(2n+2) ( 2n+2 ) !}2^{2n+2} \vert B_{2n+2} \vert \biggr) ,\quad n=1,2,\ldots . \end{aligned}$$

We now show

5.1 $$ k_{n}=- \biggl( \biggl( \frac{2}{\pi } \biggr) ^{2n+2} \frac{1}{n}-\frac{2^{2n+2}-1}{(2n+2) ( 2n+2 ) !}2^{2n+2} \vert B_{2n+2} \vert \biggr) < 0 $$

for $n\geq 1$, that is,

$$ \biggl( \frac{2}{\pi } \biggr) ^{2n+2}\frac{1}{n}- \frac{2^{2n+2}-1}{(2n+2) ( 2n+2 ) !}2^{2n+2} \vert B_{2n+2} \vert >0 $$

or

$$ \vert B_{2n+2} \vert < \frac{1}{\pi^{2n+2}}\frac{ ( 2n+2 ) !}{2^{2n+2}-1} \frac{2n+2}{n} $$

holds for $n\geq 1$. In fact, by Lemma 2.1 we have

$$ \vert B_{2n+2} \vert < \frac{2(2n+2)!}{(2\pi )^{2n+2}}\frac{2^{2n}}{2^{2n}-2}, $$

so (5.1) holds as long as we can prove that

$$ \frac{2(2n+2)!}{(2\pi )^{2n+2}}\frac{2^{2n}}{2^{2n}-2}< \frac{1}{\pi^{2n+2}}\frac{ ( 2n+2 ) !}{2^{2n+2}-1} \frac{2n+2}{n}, $$

that is,

$$ n \bigl( 2^{2n+2}-1 \bigr) < 4(n+1) \bigl(2^{2n}-2\bigr), $$

which is equivalent to

$$ 4(n+1) \bigl(2^{2n}-2\bigr)-n \bigl( 2^{2n+2}-1 \bigr) = 4 \cdot 2^{2n}-7n-8>0 $$

for $n\geq 1$. So $k_{n}<0$ for $n\geq 1$, which leads to $G_{1}^{ \prime \prime \prime }(x)=\sum_{n=2}^{\infty }2n(2n-1)(2n-2)k_{n}x ^{2n-3}<0$, and $G_{1}^{\prime \prime }(x)$ is decreasing on $(0,\pi /2)$. We can compute

$$\begin{aligned}& G_{1}^{\prime }(x) = \tan x-\frac{8}{\pi^{2}}x\ln \biggl( -4 \frac{\pi }{4x^{2}-\pi^{2}} \biggr) + \frac{32}{\pi^{2}}\frac{x^{3}}{4x^{2}-\pi^{2}} , \\& G_{1}^{\prime \prime }(x) = \tan^{2}x-\frac{8}{\pi^{2}} \ln \biggl( -4\frac{\pi }{4x^{2}-\pi^{2}} \biggr) + \frac{160}{\pi^{2}}\frac{x^{2}}{4x ^{2}-\pi^{2}}- \frac{256}{\pi^{2}}\frac{x^{4}}{ ( 4x^{2}-\pi^{2} ) ^{2}}+1, \end{aligned}$$

which give

$$ G_{1}^{\prime \prime }\bigl(0^{+}\bigr)= 1- \frac{8}{\pi^{2}}\ln \frac{4}{\pi }\thickapprox 0.80420>0, \quad\quad G_{1}^{\prime\prime} \biggl( \frac{\pi }{2}^{-} \biggr) =- \infty . $$

Then there exists an unique real number $x_{1}\in (0,\pi /2)$ such that $G_{1}^{\prime \prime }(x)>0$ on $(0,x_{1})$ and $G_{1}^{\prime \prime }(x)<0$ on $(x_{1},\pi /2)$. So $G_{1}^{\prime }(x)$ is increasing on $(0,x_{1})$ and decreasing on $(x_{1},\pi /2)$. Since

$$ G_{1}^{\prime }\bigl(0^{+}\bigr) =0,\quad\quad G_{1}^{\prime } \biggl( \biggl( \frac{\pi }{2} \biggr) ^{-} \biggr) =- \infty , $$

there exists an unique real number $x_{2}\in (x_{1},\pi /2)$ such that $G_{1}^{\prime }(x)>0$ on $(0,x_{2})$ and $G_{1}^{\prime }(x)<0$ on $(x_{2},\pi /2)$. So $G_{1}(x)$ is increasing on $(0,x_{2})$ and decreasing on $(x_{2},\pi /2)$. In view of $G_{1}(0^{+})=0=G_{1}(( \pi /2)^{-})$, the proof of the left-hand side inequality of (1.7) is complete.

(2) Let

$$ G_{2}(x)=\frac{x^{2}}{2\ln \frac{4}{\pi }}\ln \frac{4\pi }{\pi^{2}-4x ^{2}}-\ln \sec x ,\quad 0< x< \frac{\pi }{2}. $$

Then we get

$$ G_{2}(x)=\sum_{n=1}^{\infty }w_{n}x^{2n+2}, $$

where

$$ w_{n}=\frac{1}{2\ln \frac{4}{\pi }} \biggl( \frac{2}{\pi } \biggr) ^{2n} \frac{1}{n}-\frac{2^{2n+2}-1}{(2n+2) ( 2n+2 ) !}2^{2n+2} \vert B _{2n+2} \vert ,\quad n=1,2,\ldots . $$

We now show $w_{n}>0$ for $n\geq 1$, that is,

5.2 $$ \vert B_{2n+2} \vert < \frac{(n+1) ( 2n+2 ) !}{ 4n\ln \frac{4}{\pi }\pi^{2n}(2^{2n+2}-1)} $$

holds for $n\geq 1$. In fact, by Lemma 2.1 we have

$$ \vert B_{2n+2} \vert < \frac{2(2n+2)!}{(2\pi )^{2n+2}}\frac{2^{2n}}{2^{2n}-2}, $$

so (5.2) holds as long as we can prove that

$$ \biggl( 2n\ln \frac{4}{\pi } \biggr) \bigl( 2^{2n+2}-1 \bigr) < \pi^{2}(n+1) \bigl( 2^{2n}-2 \bigr) , $$

which is true for $n\geq 1$. So $G_{2}^{\prime }(x)>0$, and $G_{2}(x)$ is increasing on $(0,\pi /2)$. We can compute $G_{2}(0^{+})=0$ and $G_{2}((\pi /2)^{-})=+\infty $, the proof of the right-hand side inequality of (1.7) is complete.

(3) Let

$$ G_{3}(x)=\frac{\ln \sec x}{x^{2}\ln \frac{4\pi }{\pi^{2}-4x^{2}}},\quad 0< x< \frac{\pi }{2}. $$

Then

$$ G_{3}\bigl(0^{+}\bigr)= \frac{1}{2\ln \frac{4}{\pi }}\thickapprox 2.0698,\quad\quad G_{3} \biggl( \biggl( \frac{\pi }{2} \biggr) ^{-} \biggr) =\frac{4}{ \pi^{2}}\thickapprox 0.40528, $$

this completes the proof of Theorem 1.3.

## Proof of Theorem 1.4

Let

$$ G_{4}(x)=\frac{\frac{\ln \sec x}{\ln \frac{4\pi }{\pi^{2}-4x^{2}}}- \frac{1}{2}\frac{x^{2}}{\ln \frac{4}{\pi }}}{x^{4}}=\frac{\ln \sec x- \frac{1}{2}\frac{x^{2}}{\ln \frac{4}{\pi }}\ln \frac{4\pi }{\pi^{2}-4x ^{2}}}{x^{4}\ln \frac{4\pi }{\pi^{2}-4x^{2}}}=\frac{f(x)}{g(x)},\quad 0< x< \frac{ \pi }{2}, $$

where

$$ f(x)=p_{1}x^{4}+\sum_{n=2}^{\infty }p_{n}x^{2n+2} $$

and

$$ g(x)=q_{1}x^{4}+\sum_{n=2}^{\infty }q_{n}x^{2n+2} $$

with

$$\begin{aligned}& p_{1} =\frac{1}{12}-\frac{1}{2}\frac{1}{\ln \frac{4}{\pi }} \biggl( \frac{2}{ \pi } \biggr) ^{4}; \\ & p_{n} =\frac{2^{2n+2}-1}{(2n+2) ( 2n+2 ) !}2^{2n+2} \vert B_{2n+2} \vert - \frac{1}{2}\frac{1}{\ln \frac{4}{\pi }} \biggl( \frac{2}{\pi } \biggr) ^{2n}\frac{1}{n},\quad n\geq 2. \\ & q_{1} =\ln \frac{4}{\pi }>0; \\ & q_{n} = \biggl( \frac{2}{\pi } \biggr) ^{2n-2} \frac{1}{n-1}>0,\quad n\geq 2. \end{aligned}$$

Since

$$ \frac{p_{1}}{q_{1}}=\frac{\frac{1}{12}-\frac{1}{2}\frac{1}{\ln \frac{4}{ \pi }} ( \frac{2}{\pi } ) ^{4}}{\ln \frac{4}{\pi }}\thickapprox -1.0624 $$

and

$$ \frac{p_{n}}{q_{n}}=\frac{2 ( n-1 ) }{\pi^{2}} \biggl( \frac{4\pi^{2n}}{ ( 2n+2 ) !} \frac{2^{2n+2}-1}{n+1} \vert B _{2n+2} \vert -\frac{1}{\ln \frac{4}{\pi }} \frac{1}{n} \biggr) ,\quad n\geq 2, $$

we can obtain

$$ \frac{p_{1}}{q_{1}}\thickapprox -1.0624 < \frac{p_{2}}{q_{2}}=\frac{1}{\pi^{2}} \biggl( \frac{1}{180}\pi^{4}-\frac{1}{ \ln \frac{4}{\pi }} \biggr) \thickapprox -0.36461, $$

but

6.1 $$ \frac{p_{n}}{q_{n}}>\frac{p_{n+1}}{q_{n+1}} $$

for $n\geq 2$. The inequality (6.1) is equivalent to

$$\begin{aligned}& \frac{2 ( n-1 ) }{\pi^{2}} \biggl( \frac{4\pi^{2n}}{ ( 2n+2 ) !}\frac{2^{2n+2}-1}{n+1} \vert B _{2n+2} \vert -\frac{1}{\ln \frac{4}{\pi }}\frac{1}{n} \biggr) \\& \quad > \frac{2n}{\pi^{2}} \biggl( \frac{4\pi^{2n+2}}{ ( 2n+4 ) !}\frac{2^{2n+4}-1}{n+2} \vert B _{2n+4} \vert -\frac{1}{\ln \frac{4}{\pi }}\frac{1}{n+1} \biggr) ,\quad n\geq 2. \end{aligned}$$

By Lemma 2.1, we have

$$ \frac{2 ( n-1 ) }{\pi^{2}} \biggl( \frac{4\pi^{2n}}{ ( 2n+2 ) !}\frac{2^{2n+2}-1}{n+1} \vert B _{2n+2} \vert -\frac{1}{\ln \frac{4}{\pi }}\frac{1}{n} \biggr) > \frac{2 ( n-1 ) }{\pi^{2}} \biggl( \frac{8}{\pi^{2} ( n+1 ) }-\frac{1}{\ln \frac{4}{\pi }} \frac{1}{n} \biggr) $$

and

$$\begin{aligned}& \frac{2n}{\pi^{2}} \biggl( \frac{4\pi^{2n+2}}{ ( 2n+4 ) !}\frac{2^{2n+4}-1}{n+2} \vert B _{2n+4} \vert -\frac{1}{\ln \frac{4}{\pi }}\frac{1}{n+1} \biggr)\\& \quad < \frac{2n}{ \pi^{2}} \biggl( \frac{1}{\pi^{2} ( 2^{2n+3}-1 ) }\frac{2^{2n+6}-4}{n+2}- \frac{1}{ \ln \frac{4}{\pi }}\frac{1}{n+1} \biggr) . \end{aligned}$$

So (6.1) holds when we prove

$$ n \biggl( \frac{1}{\pi^{2} ( 2^{2n+3}-1 ) }\frac{2^{2n+6}-4}{n+2}-\frac{1}{ \ln \frac{4}{\pi }} \frac{1}{n+1} \biggr) < ( n-1 ) \biggl( \frac{8}{\pi^{2} ( n+1 ) }- \frac{1}{\ln \frac{4}{\pi }} \frac{1}{n} \biggr) , $$

or

$$ \pi^{2} \bigl( 2^{2n+3}-1 \bigr) ( n+2 ) > \biggl( \ln \frac{4}{\pi } \biggr) n \bigl( 2^{2n+7}+4n ^{2}+4n-16 \bigr) , $$

which is ensured for $n\geq 2$.

So

$$ \frac{p_{1}}{q_{1}}< \frac{p_{2}}{q_{2}}>\frac{p_{3}}{q_{3}}>\frac{p _{4}}{q_{4}}>\cdots . $$

Since

$$ H_{f,g} \biggl( \biggl( \frac{\pi }{2} \biggr) ^{-} \biggr) = \lim_{x\rightarrow (\frac{\pi }{2})^{-}} \biggl( \frac{f^{\prime }}{g ^{\prime }}g-f \biggr) =0, $$

we see that $G_{4}(x)$ is increasing on $(0,\pi /2)$ by Lemma 2.4. In view of

$$\begin{aligned}& G_{4}\bigl(0^{+}\bigr) =\alpha = \frac{1}{12\ln \frac{4}{\pi }}- \frac{2}{\pi^{2}\ln^{2}\frac{4}{\pi }} \thickapprox -3.1277, \\& G_{4} \biggl( \biggl( \frac{\pi }{2} \biggr) ^{-} \biggr) = \beta = \frac{16}{\pi^{4}} \biggl( 1-\frac{1}{8} \frac{\pi^{2}}{\ln \frac{4}{ \pi }} \biggr) \thickapprox -0.67462, \end{aligned}$$

the proof of Theorem 1.4 is complete.

## Remark

### Remark 7.1

The results of inequalities in Theorems 1.1–1.4 can be validated by methods and algorithms developed in [12, 13] and [14].
